# The Illusion of Owning a Third Arm

**DOI:** 10.1371/journal.pone.0017208

**Published:** 2011-02-23

**Authors:** Arvid Guterstam, Valeria I. Petkova, H. Henrik Ehrsson

**Affiliations:** Brain, Body and Self Laboratory, Department of Neuroscience, Karolinska Institutet, Stockholm, Sweden; City of Hope National Medical Center and Beckman Research Institute, United States of America

## Abstract

Could it be possible that, in the not-so-distant future, we will be able to reshape the human body so as to have extra limbs? A third arm helping us out with the weekly shopping in the local grocery store, or an extra artificial limb assisting a paralysed person? Here we report a perceptual illusion in which a rubber right hand, placed beside the real hand in full view of the participant, is perceived as a supernumerary limb belonging to the participant's own body. This effect was supported by questionnaire data in conjunction with physiological evidence obtained from skin conductance responses when physically threatening either the rubber hand or the real one. In four well-controlled experiments, we demonstrate the minimal required conditions for the elicitation of this “supernumerary hand illusion”. In the fifth, and final experiment, we show that the illusion reported here is qualitatively different from the traditional rubber hand illusion as it is characterised by less disownership of the real hand and a stronger feeling of having two right hands. These results suggest that the artificial hand ‘borrows’ some of the multisensory processes that represent the real hand, leading to duplication of touch and ownership of two right arms. This work represents a major advance because it challenges the traditional view of the gross morphology of the human body as a fundamental constraint on what we can come to experience as our physical self, by showing that the body representation can easily be updated to incorporate an additional limb.

## Introduction

An organism's body plan refers to the symmetry and the number of segments and limbs of the body. It is a blueprint of the morphology of the organism that is regulated by specific genes (homeogenes) during development [1]; these molecular mechanisms also specify the formation of the central nervous system [2]. It is current wisdom that the body plan imposes fundamental constraints on the neuronal representations of the body, and, thereby, on how we perceive ourselves [3]–[5]. Humans, for example, have four limbs and it is reasonable to assume that this body configuration and a life-time of experience of having such a body imposes fundamental constraints on how our perceptual systems process sensory information from it. Indeed, a familiar fact to all students of neuroscience is that afferent sensory information from the body is first processed in somatotopical maps of the body in the somatosensory cortex [6]. This map contains all limbs and body segments and is organised in an orderly fashion that corresponds to the organism's body plan across a wide range of species [7]–[11].

Interestingly, in the neurological literature, there are case reports of people with cortical or subcortical lesions who experience having extra limbs, so-called supernumerary phantom limbs. These phantoms are typically experienced as an additional arm or leg [12]–[15]. Although the mechanisms producing these body sensations are unknown [16], their existence suggests that disruption of the central circuits processing information from the body can lead to illusory sensations that violate the gross morphology of the human body plan.

From the study of body illusions in healthy individuals we know that the body representation is inherently malleable and dynamic [17]–[19]. Our perceptual systems are continuously integrating and interpreting all available sensory data to compute the spatial relationship between our limbs and other body parts. These illusions involve displacement, elongation, shrinkage, and movement of limbs and body parts, but they do not violate the basic structure of the human body plan and only work for objects that resemble human body parts [20], [21].

One such illusion, the rubber hand illusion [22], has recently become an important tool for cognitive neuroscientists to study body self-perception. In this illusion, the participant observes a rubber hand being touched in synchrony with touches applied to his or her real hand, which is hidden out of view. This creates an illusory experience that the applied touches are felt on the rubber hand and that the rubber hand is one's own. To elicit the illusion, it is crucial that the rubber hand resembles a human or primate hand of the same laterality as the hidden real hand (e.g., the real right hand and a rubber right hand) [23]–[26], that it is orientated in an anatomically plausible position in parallel with the real hand [24], [27]–[29] and that the tactile stimulation of the hands is applied synchronously [22], [24], [27] and in the same direction in hand-centred coordinates [28]. These behavioural observations suggest that the rubber hand illusion is a multisensory illusion and that the key principles determining its elicitation is the temporal and spatial congruence of the visual, tactile and proprioceptive signals in arm-centred reference frames [30], [31]. This “multisensory hypothesis of body ownership” [31] is further supported by functional magnetic resonance imaging data, which has associated activity in multisensory areas in the premotor and posterior parietal cortex with the illusory feeling of ownership [27], [32], [33].

Interestingly and intriguingly, a few recent reports have suggested that it is possible for healthy participants to experience illusory ownership of supernumerary rubber or virtual hands [34], [35]. In these experiments two visible rubber hands or virtual hands were stimulated in synchrony with touches applied to the hidden real hand, which, reportedly, produced a referral of somatic sensations to both rubber/virtual hands. These studies indicate that supernumerary limb illusions might be possible, but many important questions remain unanswered: What are the necessary and sufficient conditions for the elicitation of the supernumerary limb illusion? Is it possible to experience the illusion when one sees an artificial limb next to the normal one? And what are the consequences of the supernumerary limb illusion on the representation of the real limb?

To address these issues, we designed a novel ‘supernumerary’ version of the traditional rubber hand illusion [22]. We first demonstrate that it is possible to induce the rubber hand illusion even when the real hand is fully visible. Crucially, rather than “replacing” the real hand, the artificial hand was perceived as an extra limb without inducing significant disownership of the real hand. Secondly, we carried out additional experiments to identify the spatial and temporal principles that govern the elicitation of this ‘supernumerary hand illusion’. Finally, we compared this illusion directly to the traditional rubber hand illusion. Our results show that the supernumerary hand illusion presented here can only be induced by synchronous tactile stimulation of a person's real hand and an artificial limb which matches the former in respect of limb type (i.e., the illusion does not work with a rubber foot), laterality (i.e., left and right) and anatomical alignment. It also exhibits some qualitatively unique properties that make it different from the classical rubber hand illusion, as it involves stronger duplication of touch and ownership of two right hands, accompanied by a weaker feeling of disowning the real hand. These results are important because they demonstrate that the central nervous system, under certain conditions, when faced with two equally probable locations of a seen limb, can “split” the limb representation in two, making people experience a supernumerary limb as being part of their own body.

## Methods

### Participants

The study consists of data from five separate experiments involving a total of 154 healthy volunteers. For each experiment, we recruited a separate group of naïve participants: In the first experiment, we tested 30 individuals (20 females, mean age 25±9 years); the second one involved 44 participants (23 females, mean age 25±3 years), the third 25 (14 females, mean age 23±4 years), the fourth 26 (15 females, mean age 28±9 years) and the fifth 29 subjects (18 females, mean age 24±5 years). All participants gave their written informed consent prior to participating. The studies were approved by the Regional Ethical Review Board of Stockholm.

### Experimental setup

The experiments took place in a soundproof testing room (40 decibel noise reduction). The participants sat on a comfortable chair and rested their arms on a table in front of them. The experimenter sat opposite the participant.

**Figure 1:**
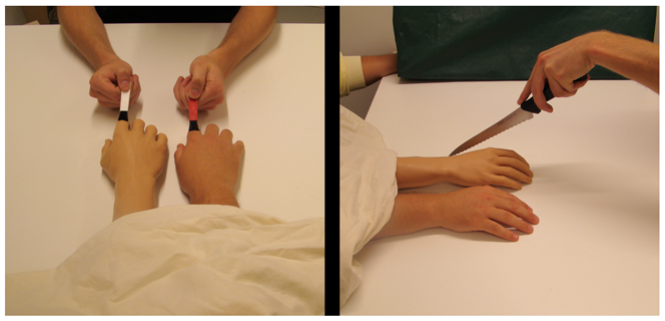
Illusion set-up. Set-up used to elicit the supernumerary hand illusion (left panel) and an illustration of the threat procedure, where we moved a knife close to the rubber hand or the real hand (right panel).

**Figure 2:**
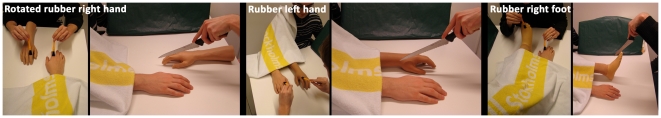
Control conditions. Set-ups used for the three different control conditions used in experiments 1, 3 and 4. The application of the brushstrokes on the real and artificial limbs is demonstrated in the left picture of each picture pair; to the right, the procedure of threatening the rubber limb with the knife is depicted. From left to right: the rotated rubber right hand, rubber left hand and rubber right foot conditions.

In all experiments and conditions the rubber hand was placed medially to the participant's real right hand (in a pilot experiment, we observed that the illusion was stronger when the rubber hand is placed medially as opposed to laterally). The distance between the index finger of the artificial limb and the index finger of the participant's right hand was 12.5 cm throughout (the only exception being in experiment 5, as described below). A piece of cloth covered the space between the participant's shoulder and the proximal end of the rubber hand. It, thus, appeared visually as if the rubber hand could be a real hand (see Figure 1). We used life-like cosmetic limb prostheses (arm or foot), which were matched to the gender of the participants (Figure 1 and 2).

The participants' left hand was placed on the table behind a screen and was, thus, hidden from view in all experiments. The participant could only see an artificial limb and their real right hand on the table, again, the only exception was in experiment 5, when a screen precluded vision of the real hand (see below).

We carefully matched the velocity, frequency and skin surface stimulated by the brushstrokes in all the experiments. The strokes were applied to the index and middle fingers of the participant's right hand and the corresponding places on the artificial limb. When a rubber right hand was used, we touched the index and middle fingers, and when a rubber foot was used, we stroked the corresponding sections on the two limbs (i.e., the index and middle finger of the participant's right hand and digits II and III on the rubber foot). When a rubber left hand or a rotated rubber right hand were used, the touches were again delivered to the corresponding digits on both hands (i.e. the index and middle fingers on the real hand and the index and middle fingers on the rubber hand).

In the illusion condition we used an irregular, but synchronous brushing rhythm, since this mode of stimulation is known to maximise the traditional rubber hand illusion (unpublished observations). In the asynchronous (control) condition, the pattern of brushing was irregular and alternating between the real hand and the rubber limb, which is an established method to break down and control for the illusion [22], [24], [27]. In all conditions, the participants were told to look at the brushstrokes on the artificial limb throughout the stimulation session. The direction of the participants' gaze was carefully controlled by the experimenter approximately every 10 seconds by looking at their eyes.

### Questionnaires: subjective measures of the illusion

To quantify the perceptual experiences associated with the illusion, we used questionnaires with visual analogue rating scales which were presented at the end of each condition. The questionnaires were adapted from a previous study investigating perceptual experiences during the traditional rubber hand illusion [22]. In the questionnaire, the participants were asked to affirm or deny ten possible perceptual effects using a ten-point visual analogue scale ranging from 0 to 9. The participants were informed that 0 meant “I do not agree at all” and 9, “I agree completely”. Two statements were used to capture the key perceptual components of the illusion of owning the rubber hand (S1–S2); two statements were created to describe the possible experience of disowning the real (right) hand (S3–S4); and two statements where formulated to capture the illusion of owning two right hands (S5–S6). The last four statements served as controls for suggestibility and task-compliance (S7–S10). For each participant we clarified that the formulation “both hands” means “the rubber limb and your real right hand” to ensure that they understood which two limbs we were referring to in our statements.

### Skin conductance response (SCR): objective physiological measures of the illusion

We used the procedure of physically threatening the rubber limb or the real hand with a knife and measure the brief increases evoked in the conductance of the skin to provide objective evidence for the illusion. The SCR reflects “psychologically” increased sweating attributable to the activation of the autonomic nervous system [36]. When one's body is physically threatened, the SCR can be used as an index of fear and pain anticipation. This has been shown to be a reliable index of illusory body ownership [32], [34], [37]–[39].

In the present study we threatened the artificial limb (experiments 2 and 4) and the person's real right hand (experiment 2) with a knife after a period of repeated brushing of the two hands. We always included appropriate control conditions (see the next section) and could therefore relate changes in the SCR to changes in ownership, excluding more general factors such as surprise, general arousal, or unspecific emotional responses related to the presentation of the knife.

The SCR were recorded with a Biopac System MP150 (Goleta, USA) following standard published guidelines [36]. Two electrodes were attached to the distal phalanges of the index and middle fingers of the participants' left hand. We used Biopac's isotonic gel (Gel 101) to ensure good contact, and the participants wore the electrodes for a few minutes before the recording was initiated. The data was registered at the sample rate 100 Hz and processed with the manufacturer's software AcqKnowledge 4.0 for Windows. The parameters of the recording were as follows: The gain switch was set to 5 µmho/V and the value of the CAL2 scale was set to 5. The timing of the threat events was indicated in the raw data files during the recordings by the experimenter pressing a key.

The knife-threat procedure consisted of moving a kitchen knife slightly above (1–2 cm) the thumb and index finger of the participant's hand or the rubber hand (experiments 2 and 4, see below), or above digits I and II on the rubber foot (experiment 4, see below). The visible movement of the knife took approximately one second and was performed so that the knife was always moved along the sagittal axis from the participant's point of view. Great care was taken to perform the same movement with the knife from trial to trial, i.e. controlling the velocity and acceleration of the movement. Before commencing the experiments, the participants were instructed to stop looking at the rubber hand and start looking at the knife as soon as it was presented to make sure that they perceived the threat. The threat-evoked SCR was identified as the peak in the conductance that occurred up to 5 seconds after the onset of the threat stimuli (that is from the first moment the participant saw the knife approaching one of the hands), which was flagged in the SCR recording file. The amplitude of the SCR was calculated as the difference between the maximum and minimum value of the identified response. This analysis was conducted with the scientist performing the analysis being blinded to whether the data in question belonged to the illusion or a control condition. The average of all responses for a participant, including those where no increase in amplitude was apparent, was calculated for each condition separately, and constituted the magnitude of the SCR [36]. Thereafter, the SCR magnitudes of all participants were compared statistically across different conditions in experiments 2 and 4, as described in the next section. Participants who did not display any threat-evoked SCR in more than half of the trails were excluded from the analysis [20].

### Experimental design: rationale and conditions

The first two experiments sought to establish the supernumerary limb illusion and to present subjective (experiment 1) and physiological evidence (experiment 2) substantiating the effect. In experiments 3 and 4, we again used subjective and objective measures to examine which type of limb could be owned as a supernumerary limb. Finally, the fifth experiment compared the perceptual experiences during the supernumerary hand illusion and the traditional rubber hand illusion.

All experiments consisted of multiple experimental conditions, which were motivated by the specific hypotheses tested (see below); the order of the stimulation conditions was semi-randomised and balanced across participants in all experiments. Additionally, in the SCR experiments, the target of the physical threat (real or artificial limb) was balanced across sessions and participants (in experiments 2 and 4).

#### Experiment 1 Introspective evidence for the supernumerary hand illusion

In the first experiment we quantified the perceptual experiences associated with the supernumerary hand illusion. In addition, we examined the possible, simultaneous feeling of disownership of the real hand. Specifically, we tested our prediction that the elicitation of the illusion would depend on the synchronicity of the seen and felt touches, and on the anatomical alignment between the owned rubber hand and the real hand. These factors are known to be crucial for the induction of the traditional rubber hand illusion [22], [24], [27]–[29], [37]. Thus there were three different conditions: the synchronous and asynchronous conditions, with the rubber hand aligned parallel to the participant's real hand, and one condition of synchronous brushing where the rubber hand was rotated through 180 degrees (representing the illusion and two control conditions). The synchronous brushstrokes in the rotated condition were applied in the same direction on the rubber hand and the real hand in external world coordinates, i.e., when we stroked to real hand from the distal part of a digit to the proximal part we stroked the rubber hand from the proximal part to the distal part. The three different conditions were tested in three separate two minute long periods of brushing. At the end of each brushing session, the participants were presented with the questionnaire and asked to fill it in.

#### Experiment 2 Physiological evidence for the supernumerary hand illusion

The aim of the second experiment was two-fold. First, to present objective physiological evidence for the illusion, and second, to examine possible disownership of the real right hand. The experiment consisted of two sessions, each divided into four, one-minute-long periods of synchronous or asynchronous brushing of the participants' real right hand and a rubber right hand. In this experiment, the rubber hand was always oriented in an anatomically congruent position parallel to the real hand. At the end of each stimulation period, the participants observed the scientist holding a knife and moving it towards and above the rubber hand or the real hand as if attempting to cut the hand in question (as described in the paragraph on SCR).

#### Experiment 3 Introspective evidence for what type of limb that can be owned as a supernumerary limb

Next we investigated which types of limb can be experienced as a supernumerary limb. We hypothesised that they would have to resemble the real stimulated hand in both laterality (e.g., both right hands) and anatomy (i.e., both arms, but not an arm and a leg). For the elicitation of the traditional rubber hand illusion, it is known to be crucial that the rubber hand resembles a human or primate hand [23]–[26] of the same laterality as the hidden real hand [23]–[26]. Thus we compared synchronous stimulation of the real right hand and a rubber right hand (illusion condition), a rubber left hand or a rubber right foot (control conditions). The three different rubber limbs were tested in three separate two minute long periods of synchronous brushing, each being followed by the presentation of the same questionnaire we used in experiment 1 to assess the subjective experiences of the illusion. All other procedures followed those of the first experiment (described above).

#### Experiment 4 Physiological evidence for what type of limb that can be owned as a supernumerary limb

In this experiment we used physiological data (SCR) to complement and support the findings in experiments 1 and 3 that only a rubber right hand can be experienced as a supernumerary limb located next to the real right hand. In addition, we tested the hypothesis that the rubber right hand has to be placed in an anatomically plausible orientation with respect to the body and the real hand for the illusion to work.

This experiment comprised three sessions, each consisting of four conditions of one minute duration, as follows: a rubber right hand aligned parallel to the participant's real right hand, a rubber right hand rotated through 180 degrees, a rubber left hand orientated parallel to the participant's real right hand and a rubber right foot aligned parallel to the participant's real right hand. In all conditions we used a synchronous mode of brushing. The rubber foot was fixed in a position with 20 degrees plantar flexion in the ankle joint to make the brushing procedure easier for the experimenter (see Figure 2, the two pictures on the right). The distance between the index finger of the participant's real hand and digit II on the rubber foot was always 12.5 cm, measured horizontally. At the end of each stimulation period, the participants observed a knife approaching and sliding over the rubber hand or foot in a “cutting” motion following the procedures described in experiment 2.

#### Experiment 5 The unique qualities of the supernumerary hand illusion

In our final experiment, we tested whether the present supernumerary hand illusion is qualitatively different from the traditional rubber hand illusion. Thus we conducted a questionnaire-based experiment comparing two conditions: synchronous brushing of a rubber right hand and of the participant's real right hand when the latter was either fully visible (supernumerary hand illusion) or hidden from view (rubber hand illusion). If, as hypothesised, the present illusion is not merely a weak form of the rubber hand illusion, one would expect a difference in the degree of disownership of the real right hand and a stronger feeling of having two right hands during the supernumerary hand illusion.

**Figure 3:**
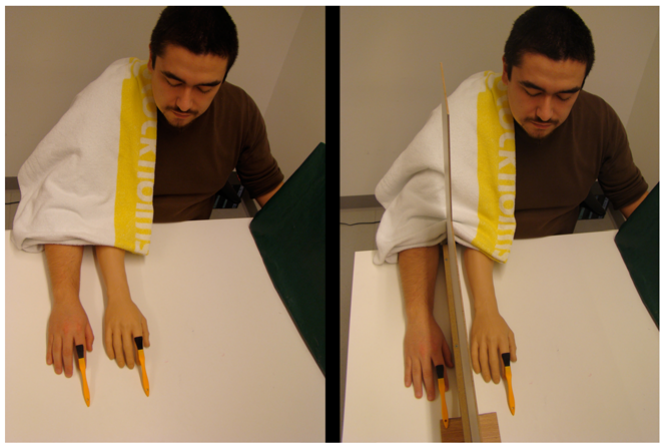
Supernumerary hand illusion vs. traditional rubber hand illusion. Set-up used in experiment 5 to elicit the supernumerary hand illusion (left panel) and the traditional rubber hand illusion (right panel). Note that the only difference was the screen occluding vision of the real right hand in the latter condition.

In this experiment we used the same general procedures as in experiments 1–4, with some important modifications. In the classical rubber hand illusion condition, we used a screen to separate the rubber and the real hands to obscure the real hand from the participant's field of vision (see Figure 3). Owing to the thickness of the screen, the distance between the index fingers of the rubber hand and the real hand was changed to 13 cm for females and 15 cm for males (depending on the different size of the rubber male and female hands). With the exception of this latter small modification, the supernumerary illusion condition was identical to the synchronous condition used in experiments 1–4. Thus the experiment consisted of two, two-minute-long periods of synchronous brushing, each followed by the presentation of the same questionnaire as used in experiments 1 and 3.

### Statistical analysis

A Kolmogorov-Smirnov test was used to check the parametric assumptions of the data in all experiments. For all ANOVAs with a repeated measures factor, we performed Mauchly's sphericity test for compound symmetry. When compound symmetry was not satisfied (as was the case in the analysis of statement S1–S2 and S3–S4 in experiment 1 and the analysis of experiment 4) we used the Huynh-Feldt epsilon-corrected degrees of freedom. In all of the tests, alpha was set to 5%. We inverted the scores of S3 to facilitate the statistical analysis of S3–S4 in the questionnaires, since low ratings on S3 and high ratings on S4 reflect stronger feelings of real hand disownership.

## Results

### Experiment 1 Introspective evidence for the supernumerary hand illusion

**Figure 4:**
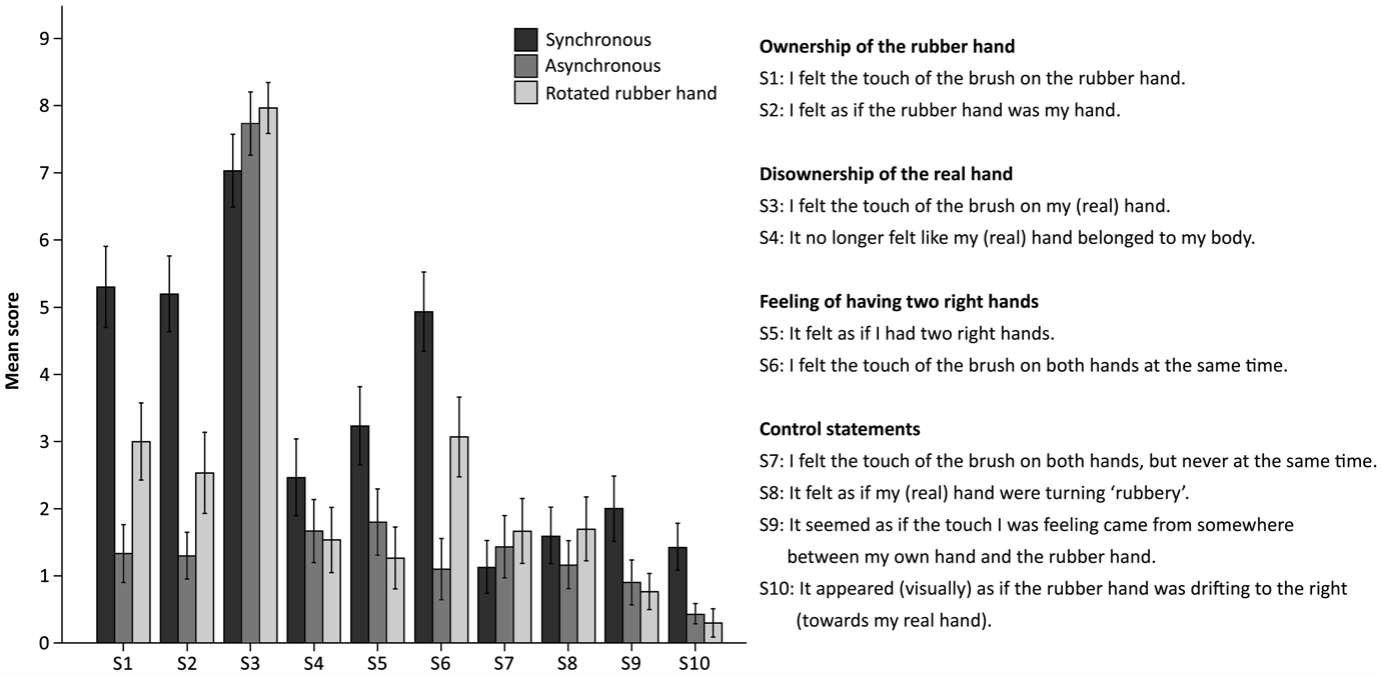
Introspective evidence for the supernumerary hand illusion. Questionnaire data from experiment 1 comparing synchronous brushing (the illusion condition) with asynchronous brushing and using a rotated rubber hand (the control conditions). The questionnaire consisted of ten statements, S1–S10, and the participants indicated their responses on a ten-point visual analogue scale ranging from 0 (“I do not agree at all”) to 9 (“I agree completely”). We observed significantly stronger rubber hand ownership (S1–S2) (p<0.001 and p<0.001, respectively), no significant difference in real hand disownership (S3–S4) (p = 0.137 and p = 0.063, respectively) and a significantly stronger feeling of owning two right hands (S5–S6) (p<0.001 and p = 0.002, respectively) during the synchronous condition compared to the asynchronous and rotated rubber hand control conditions, respectively. The error bars represent the standard error.

The results in the first experiment demonstrate that the illusion of owning a supernumerary hand requires synchronous visuo-tactile stimulation and a rubber hand orientated in parallel with the real hand. As can be seen in Figure 4, the participants reported a greater feeling of ownership (S1–S2) and of having two right limbs (S5–S6) and sensing touches both on the rubber hand and the real hand (S3–S4) in the synchronous condition than in the two control conditions, using asynchronous stimulation and a rotated rubber hand.

In the statistical analysis we performed three separate 2×3 repeated measures ANOVAs with the main factors being Condition (Synchronous, Asynchronous, Rotated) and Statement. In the first ANOVA, the Statement factor corresponded to the pair of illusion-related statements reflecting “rubber hand ownership” (S1, S2); in the second ANOVA it was the pair reflecting “real hand disownership” (S3, S4), and in the third one it was the statements related to “the feeling of having two right hands” (S5, S6). We calculated the main effects of the factor Condition for each pair of statements, and the simple contrasts between the levels of Condition (using Synchronous as the reference level) to compare the illusion to each of the control conditions individually. The analysis of S1 and S2 demonstrates that the participants felt significantly stronger ownership of the rubber hand in the synchronous condition than in the asynchronous and rotated ones, respectively (F(1, 29)  = 44.811, p<0.001 and F(1, 29)  = 16.395, p<0.001, simple contrasts). There was no significant difference in the perception of disowning the real hand during the illusion compared to the control conditions (S3–S4) (F(1, 29)  = 2.337, p = 0.137 and F(1, 29)  = 3.727, p = 0.063, simple contrasts, respectively). We obtained significantly higher ratings for the statements related to the feeling of owning two right hands during the illusion than in the control conditions (S5–S6) (F(1, 29)  = 18.065, p<0.001 and F(1, 29)  = 11.879, p = 0.002, simple contrasts, respectively). It can also be pointed out that the main factor Condition was significant for the “rubber hand ownership” (S1–S2) (F(2, 58)  = 22.529, p<0.001) and the “two hand ownership” (S5–S6) (F(2, 58)  = 11.339, p<0.001), but not for the “disownership” statements (S3–S4) (F(2, 58)  = 2.071, p = 0.135).

Next, we compared the illusion-related statements and the control statements, and found that the difference between the illusion statements and control ones was greatest in the synchronous conditon (the statistical analysis can be found in Text S1 in Supporting Information).

Finally, we compared the scores of S6 (“I felt the touch of the brush on both hands at the same time”) and S7 (“I felt the touch of the brush on both hands, but never at the same time”) to make a direct test of whether the participants experienced genuine duplication of touch and were not merely switching between feeling touch on and owning one of the right hands at a time. For this planned comparison, we conducted a two-way paired samples t-test of the scores in the synchronous (illusion) condition only. This revealed a significant difference (N = 30, p<0.001) indicating that the participants did not experience a switch in the ownership of one hand or the other alternately, but a genuine sensation of duplication of touch and ownership of two right hands. This result is reproduced two more times in this study (see experiments 3 and 5).

### Experiment 2 Physiological evidence for the supernumerary hand illusion

**Figure 5:**
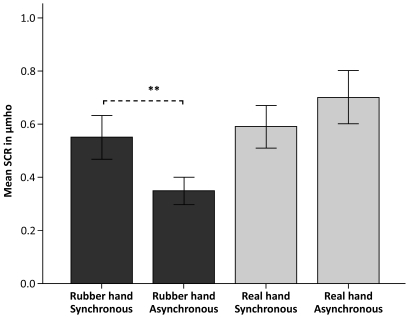
Physiological evidence for the supernumerary hand illusion. Physiological data from experiment 2 showing the mean skin conductance response (SCR) for 44 participants when the real hand or the rubber hand was threatened during synchronous brushing (the illusion condition) or asynchronous brushing (the control condition). There was a significantly greater SCR when threatening the rubber hand during the illusion (p = 0.001), but no significant difference in the SCR when threatening the real hand during the illusion compared to the control condition (p = 0.534). Thus, participants experienced ownership of the rubber hand without disowning their real hand. The error bars represent the standard error.

The key observation was a significantly greater SCR when we threatened the rubber hand in the synchronous condition than in the asynchronous one (N = 44, p = 0.001, two-tailed paired samples t-test) (Figure 5). This provides objective evidence for the ownership of the rubber hand in the present supernumerary set-up. Furthermore, and consistent with the results obtained in experiment 1, we observed no effect of disownership of the real hand as evident from the insignificant difference in the SCR between the synchronous and asynchronous control conditions when the real hand was threatened (N = 44, p = 0.534, two-tailed paired samples t-test).

We also employed a 2×2 repeated measures ANOVA to the data. The effect for the main factor, Timing (Synchronous, Asynchronous), was not significant (N = 44, F(1, 43)  = 2.099, p = 0.155) however the main factor, Hand (Rubber, Real), was (N = 44, F(1 43)  = 10.912, p = 0.002), which implies that the participants were significantly more afraid when their real hand was threatened. Crucially, there was a significant interaction Timing × Hand (N = 44, F(1, 43)  = 8.111, p = 0.007), meaning that the effect of the synchronicity of the brushing was greater for the rubber hand than the real hand. These physiological recordings, in conjunction with the subjective reports in experiment 1 and spontaneous comments made after the experiments, suggest that the participants experienced their real hand as their own, while simultaneously experiencing ownership of the rubber limb.

### Experiment 3 Introspective evidence for what type of limb that can be owned as a supernumerary limb

**Figure 6:**
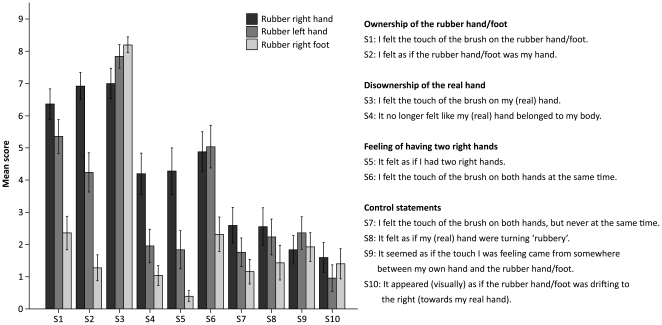
Introspective evidence for what type of limb that can be owned as a supernumerary limb. Questionnaire data from experiment 3 where synchronous brushing was applied on the real right hand and a rubber right hand (the illusion condition), a rubber left hand or a rubber right foot (the control conditions). During the illusion, we observed significantly stronger rubber hand ownership (S1–S2) compared to the rubber left hand (p = 0.007) and rubber right foot conditions (p<0.001), respectively; significantly stronger real hand disownership (S3–S4) than for both the rubber left hand (p<0.001) and the rubber right foot conditions (p<0.001); and a significantly stronger feeling of having two right hands (S5–S6) compared to the rubber right foot condition (p<0.001). This implies that participants only experience ownership of a supernumerary rubber hand when it resembles the real hand in respect to laterality (i.e. right-left matching) and limb type (i.e. both hands, but not a hand and a foot). The error bars represent the standard error.

The results of the third experiment demonstrated that the supernumerary limb illusion only worked when a rubber right hand was used (in conjunction with the real right hand) as the illusion was significantly reduced when it was replaced by a rubber left hand or right foot (Figure 6). As in the analysis for experiment 1, we performed three separate 2×3 repeated measures ANOVAs, but here we used the main factors Limb (Rubber right hand, Rubber left hand, Rubber right foot) and Statement. In the first ANOVA, the Statement factor reflected the pair of statements corresponding to the illusion of “rubber hand ownership” (S1, S2), in the second ANOVA it was the pair reflecting “real hand disownership” (S3, S4), and in the third it was the statements related to the “feeling of having two right hands” (S5, S6). We calculated the main effects of the factor Limb for each pair of statements, and the simple contrasts between the levels of Limb (using Rubber right hand as the reference level) to compare the illusion to each of the control conditions individually. For the main factor Limb the “rubber hand ownership” (S1–S2) (F(2, 48)  = 39.150, p<0.001), the “real hand disownership” (S3–S4) (F(2, 48)  = 18,400 p<0.001) and the “two hand ownership” (S5–S6) statements (F(2, 48)  = 15.462, p<0.001) were all significant. Importantly, the participants agreed significantly more strongly on the questionnaire statements relating to rubber hand ownership (S1–S2) during the rubber right hand condition (i.e., the illusion condition) in comparison to the rubber left hand and the rubber right foot conditions, respectively (S1–S2) (F(1, 24)  = 8.594, p = 0.007 and F(1, 24)  = 106.457, p<0.001, simple contrasts).

We also, somewhat surprisingly since we did not see this in Experiment 1–2, observed significantly stronger ratings for the disownership of the real hand (S3–S4) during the illusion condition than for both of the control conditions (S3–S4) (F(1, 24)  = 21,573, p<0.001 and F(1, 24)  = 24,808 p<0.001, simple contrasts). Lastly, for the statements related to the feeling of having two right hands (S5–S6), the ratings were significantly higher in the illusion condition than for the rubber right foot control condition (S5–S6) (F(1, 24)  = 26.810, p<0.001, simple contrast). Unexpectedly, this was not significant for the comparison between the illusion condition and the rubber left hand control condition (S5–S6) (F(1, 24)  = 3.110, p = 0.091, simple contrast). This was due to the high ratings for the “duplication of touch statement” (S6) for the rubber left hand (Figure 6). Post-hoc analysis revealed that the experienced duplication of touch did not significantly differ when the rubber hand was a right or a left hand (S6) (N = 25, p = 1.0, two-tailed paired samples t-test, Bonferroni correction) while the feeling of owning two right hands was significantly stronger in the rubber right hand condition (S5) (N = 25, p = 0.006, two-tailed paired samples t-test, Bonferroni correction).

Next, we compared the illusion-related statements and the control statements, and found that the difference between the illusion statements and control ones was greatest in the synchronous conditon (the statistical analysis can be found in Text S2 in Supporting Information).

A direct contrast of S6 and S7 was made to determine whether participants tended to experience two separate touches on both hands rather than merely switching between feeling touches on one hand or the other alternately. For this, we used a two-way paired samples t-test on the scores in the rubber right hand condition. We found a significant difference (N = 25, p = 0.025), in good agreement with experiment 1, confirming that people experienced duplication of touch.

### Experiment 4 Physiological evidence for what type of limb that can be owned as a supernumerary limb

**Figure 7:**
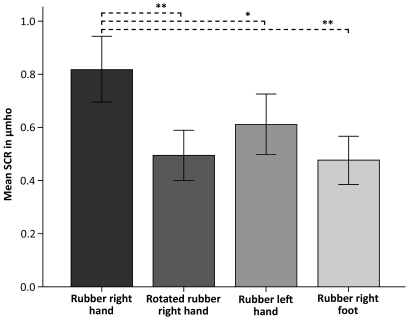
Physiological evidence for what type of limb that can be owned as a supernumerary limb. Physiological data from experiment 4 showing the mean SCR for 26 participants when the artificial limb was threatened during the illusion (rubber right hand) and three control conditions (rotated rubber right hand, rubber left hand and rubber right foot). Planned comparisons revealed significantly greater SCR when threatening the artificial limb during the illusion compared to each of the three control conditions involving a: Rotated rubber right hand (p = 0.001), rubber left hand (p = 0.048), rubber right foot (p<0.001), respectively. Thus, these results provide SCR evidence that the rubber limb needs to resemble the real limb in respect of anatomical alignment, laterality and limb type for the supernumerary limb illusion to arise. The error bars represent the standard error.

We observed greater threat-evoked SCR in the illusion condition (i.e. when we used a rubber right hand) than in the three control conditions conducted with a rotated rubber right hand, a rubber left hand or a rubber right foot (N = 26, F(3, 75)  = 7.452, p<0.001, repeated measures one-way ANOVA) (Figure 7). Planned comparisons revealed a significant difference between the conditions rubber right hand vs. rotated rubber right hand (N = 26, p = 0.001, paired two-tailed t-test), rubber right hand vs. rubber left hand (N = 26, p = 0.048) and rubber right hand vs. rubber right foot (N = 26, p<0.001, paired two-tailed t-test), implying that people felt significantly stronger ownership of the rubber limb during the illusion than in the control conditions.

### Experiment 5 The unique qualities of the supernumerary hand illusion

**Figure 8:**
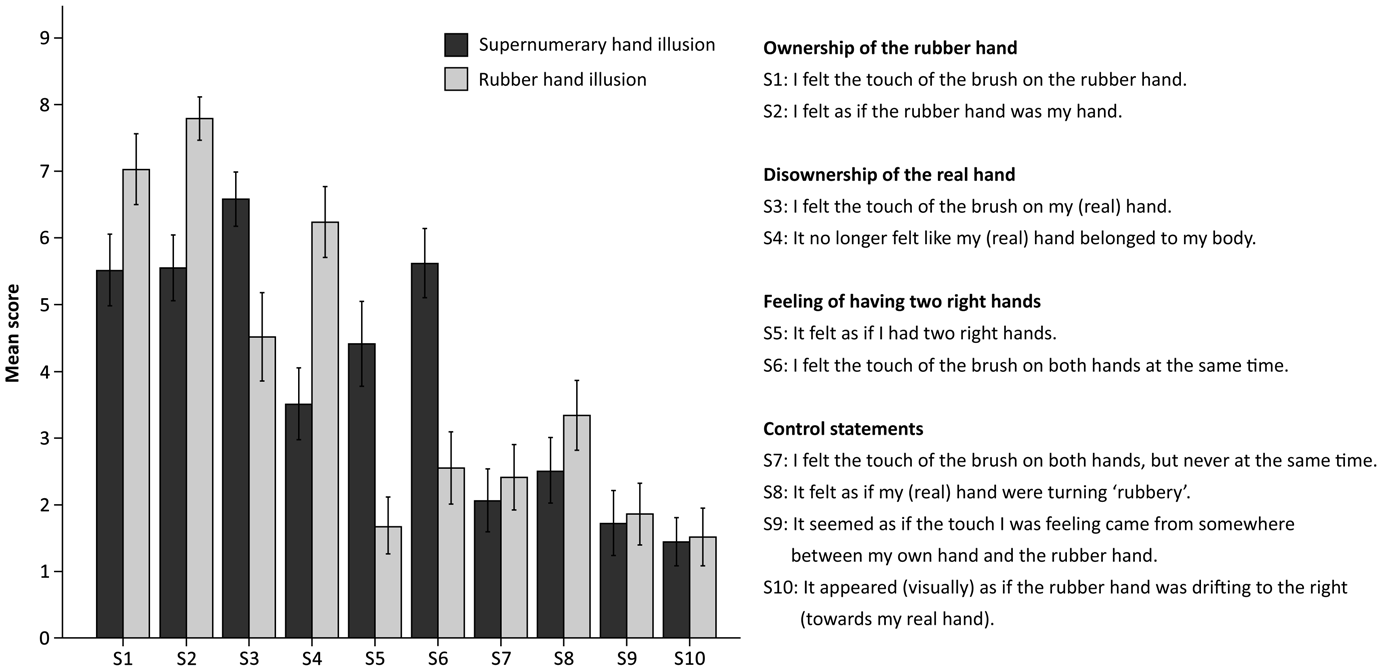
The unique qualities of the supernumerary hand illusion. Questionnaire data from experiment 5 demonstrating the differences between the supernumerary hand illusion and the traditional rubber hand illusion. During the supernumerary hand illusion, participants experienced significantly weaker rubber hand ownership (S1–S2) (p<0.001), less real hand disownership (S3–S4) (p = 0.001) and had a stronger feeling of having two right hands (S5–S6) (p<0.001). The error bars represent the standard error.

In this experiment, we compared the supernumerary hand illusion directly with the traditional rubber hand illusion to test our hypothesis that the two perceptual illusions are qualitatively different (see Figure 8). In so doing, we employed three 2×2 repeated measures ANOVAs with the factors Illusion type (Supernumerary hand illusion, Rubber hand illusion) and Statement. As in experiments 1 and 3, in each ANOVA, one of the three pairs of illusion statements constituted the factor Statement: “rubber hand ownership” (S1, S2), “real hand disownership” (S3, S4) and “feeling of having two right hands” (S5, S6), respectively. The results revealed that, during the supernumerary hand illusion, the participants experienced significantly weaker ownership of the rubber hand (S1–S2) (F(1, 28)  = 18.056, p<0.001, main effect of Illusion type), significantly less disownership of their real right hand (S3–S4) (F(1, 28)  = 15.108, p = 0.001, main effect of Illusion type) and a significantly stronger feeling of owning two right hands (S5–S6) (F(1, 28)  = 21.478, p<0.001, main effect of Illusion type) in comparison to the rubber hand illusion.

As expected, both illusions produced greater scores for the illusion-related statements than the control questions (i.e. a significant effect of the main factor Statement type (F(1, 115)  = 122.107, p<0.001). In other words, they both represent genuine perceptual phenomena that cannot be explained in terms of suggestibility, imagination, or task compliance. Interestingly, neither the main factor Illusion type (F(1, 115)  = 0.0105, p = 0.747), nor the interaction Illusion type × Statement type (F(1, 115)  = 3.438, p = 0.066) were significant, implying that the “total amount of illusory experience” did not significantly differ between the supernumerary hand illusion and the traditional rubber hand illusion.

It was also interesting to see if the two illusions would differ in terms of how the duplication of touch on the two hands was perceived. We examined this by comparing the rating scores of S6 and S7 for the two conditions. For the third time, for the supernumerary hand illusion, we were able to reproduce the significant difference between the ratings of the two statements (N = 29, p<0.001, paired two-tailed t-test) (see Experiments 1 and 3). Thus, in this illusion, people have a strong sensation that the single tactile stimulation on the real hand is duplicated and felt on the two hands simultaneously. In striking contrast, no such significant difference was observed in the rubber hand illusion condition (N = 29, p = 0.839, paired two-tailed t-test). This is consistent with the fact that people tend to experience touch as being located mainly on the rubber hand in the classical rubber hand illusion. This observation again reinforces the qualitative differences between the present illusion and the original rubber hand illusion.

## Discussion

The results of our experiments demonstrate that people can have the experience that an artificial hand is a supernumerary limb belonging to their own body. This perceptual illusion arises when a rubber hand is placed beside the participant's real hand in full view and both hands are brushed on corresponding sites in a synchronous manner. This study identifies four factors which are necessary to elicit the illusion on the right hand: the rubber limb must be matched to the real limb in terms of (i) laterality; (ii) limb type (i.e. the illusion does not work with a rubber left hand or a rubber foot); (iii) anatomical alignment (the rubber hand must be placed in an anatomically congruent position with respect to the real one and the person's body); and (iv) the visual stimulation on the rubber hand and the visuo-tactile stimulation on the real hand must be synchronous. Taken together, these results indicate that ownership of the supernumerary hand depends on achieving a match between the visual information about the spatial orientation of the rubber hand and proprioceptive information about the orientation of the real hand, and on a match between the correlated visual and tactile information from the two hands. These factors bear striking similarities with the traditional rubber hand illusion and suggest that the supernumerary hand ‘borrows’ some of the multisensory processes normally used to identify and localise the real limb [30], [31]. However, the supernumerary hand illusion is not merely a rubber hand illusion with the real hand being visible: The former is characterised by a stronger feeling of owning two hands simultaneously, a greater sense of duplication of touch, less ownership of the rubber hand, and less disownership of the real hand, suggesting that it represents a different perceptual phenomenon. Taken together, these findings are important because they challenge the traditional view of the gross morphology of the human body as a fundamental constraint for own-body perception, and instead suggest a highly flexible model of the body representation which can be reshaped to include an extra limb.

What neural mechanisms might be responsible for the present supernumerary limb illusion? According to the multisensory hypothesis of body ownership [31], the self attribution of a limb is the result of a binding of visual, tactile and proprioceptive information in limb-centred reference frames [22]–[24], [27], [40], [41]. Input from these three sensory modalities converge to multisensory areas of the cortex, including neuronal populations in the premotor and posterior parietal cortices [23], [27], [40], [42]. Through multisensory integration performed by neurons in these areas, the different sources of sensory information are combined to define what objects in our ‘near-body space’ that belong to the own body, and which objects that belong the external environment [18], [30], [31]. How can we understand the present supernumerary hand illusion within this multisensory framework? The key aspect of our illusion is that there are two possible solutions to the multisensory correspondence problem (or the ‘assignment problem’) [43], [44]. Sensory evidence is presented favoring the interpretation that each of the two hands is one's own hand. Interestingly, rather than choosing one solution, the brain seems capable of accepting two equally likely solutions at the same time, leading to simultaneous self-identification of both right arms. This phenomenon is probably best understood in a probabilistic framework of multisensory integration [43]–[46]. In this view, the nervous system encodes the location of the right arm using probabilistic population codes allowing for biphasic probability distributions [47]. Thus, when seeing one's own hand and a rubber hand receiving matching visuo-tactile stimulation, multisensory integration processes in the brain presumably calculate two equally probable locations of the hands and touches. At the neuronal level one could speculate that the multisensory neurons in the premotor and posterior parietal cortices [23], [27], [40], [48] split up into two sub-populations, each representing the arm-centred coordinates of one of the right limbs. A prediction of this model was that the experience of ownership of two right hands would add up to the total ownership of a single right hand [34]. Although one can see a trend in this direction in the SCR data of experiment 2 (see Figure 5), the ownership of the rubber hand was not accompanied by significant disownership of the real hand. Nevertheless, the results of experiment 5 seem more compatible with this prediction. Here we observed significantly weaker ownership of the rubber hand in combination with weaker real hand disownership when comparing the supernumerary and the traditional rubber hand illusions, with no significant differences in the overall rating scores across the illusion-related statements. Thus, we propose that the illusion of owning a supernumerary right arm is a perceptual phenomena which arises at level of multisensory integration in the brain, and the “neural sub-population model” presented here can presumably be tested in brain imaging studies of the illusion.

Why is the real right hand not disowned, as one could reasonably expect if the rubber hand utilises some of the same multisensory processes used to localise and identify the real hand? Although we observed significant disownership in the questionnaire in experiment 3, this was effect was not seen in the questionnaire data in experiment 1 or when analysing the threat-evoked SCR in experiment 2. Thus, even though it is possible that more sensitive methods could discover subtle disownership-related effects in the present set-up, overall, the emerging picture is one of strong ownership of the real hand in the presence of ownership of the rubber one, which is what one could reasonably refer to as a supernumerary hand illusion. It is noteworthy that the threats to the real hand always produced greater a SCR than threats to the rubber one (experiment 2) further confirming that the real hand was still perceived as one's own.

These considerations raise an interesting question: Why is the ownership of the real hand so resistant to the supernumerary hand illusion? During this illusion, participants receive visual information about their real right hand in the periphery of their field of vision. The questionnaire results from experiment 5 demonstrate that this hand is disowned to a significantly lesser degree during the supernumerary hand illusion presented here than in the traditional rubber hand illusion where the participant is deprived of visual information from the real hand. Thus, despite experiencing ownership of a rubber right hand, the representation of the real hand is seemingly not impaired because of the visual impression of it being brushed.

In experiment 3 we compared the supernumerary illusion with a rubber right hand to a control condition with a rubber left hand. As expected, in the rubber left hand condition people did not experience ownership of the hand, but somewhat surprisingly they reported a rather high degree of touch duplication. It is interesting here to compare these results with a recent study by Petkova and Ehrsson (2009). These authors described an illusion where participants were exposed to synchronous tactile stimulation of a rubber right hand and their real left hand (the unstimulated real right hand being parallel to the rubber right hand and hidden behind a screen), which induced ownership of the rubber right hand and a duplication of touch from the real left hand to the rubber right hand “across the body midline” [38]. In our experiments, the real left hand was always located on the table hidden from view (parallel to the rubber left hand; see Figure 1 left panel). Thus, the only important difference between Petkova's illusion setup and our “rubber left hand” condition is the distance between the stimulated hands (42 cm and 12.5 cm). Importantly, however, this experimental manipulation resulted in marked perceptual differences. In the set-up reported here, the visual impression of the rubber left hand being on the right next to the real right hand was inconsistent with the position sense information from the hidden real left hand, so the full illusion of ownership was not triggered. In contrast, in Petkova and Ehrsson's setup, the rubber right hand matched the felt orientation of the hidden real right hand, causing illusory rubber hand ownership of the right hand and duplication of touch from the real left hand to the rubber right one, probably involving activation of somatosensory neurons with bilateral receptive fields. The surprisingly high degree of touch duplication from the real right hand to the rubber left one in the experiment reported here could be explained by a partial engagement of the same bilateral somatosensory mechanism as in Petkova and Ehrsson's bilateral rubber hand illusion.

The study presented here differs in several important ways from previous ones on supernumerary limb illusions in healthy individuals [34], [35], [49]. In Ehrsson (2009), two rubber right hands were presented while the participant's real hand was hidden under the table. In Newport et al. (2009) the participants viewed two video-images of their real left hand, i.e. they observed two copies of their real left hand with the help of video-technology. In these earlier experiments it was thus uncertain if a third arm was owned while maintaining ownership of the real arm, and if the visual duplication of the rubber/video hand created a genuine duplication of the limb or merely produced a weak rubber hand illusion on both of the visible hands, where the participant switched between owning one of the two at a time. The present data represent a more convincing demonstration of a supernumerary hand illusion, where the real hand is fully visible and owned, as we could prove that both hands were owned simultaneously and demonstrate differences from the traditional rubber hand illusion. Furthermore, in contrast to the systematic set of experimental manipulations used here to identify the factors that are critical to elicit the illusion, Ehrsson (2009) only used synchronous and asynchronous conditions and Newport et al. (2009) only a synchronous condition, with the control being the presentation of a single video image of the hand.

In Schaefer et al. (2009), participants observed a rubber left hand that had been placed next to their visible real left hand. No synchronous stroking of the hands was employed, making it questionable if an ownership-illusion was ever produced. This concern is further strengthened by the low scores given by the subjects when asked to rate two illusion-statements [49]. Indeed, it is known that the presentation of a rubber hand without synchronous visuo-tactile stimulation does not elicit the rubber hand illusion [50]. This concern makes it hard to interpret the functional meaning of the changes in dipole location in the primary somatosensory cortex, which Schaefer and colleagues observed using magnetic source imaging when stimulating the thumb.

Our results have bearing on the emerging research field of neuroprosthetics. Over the last decade, a tremendous effort has been made to design brain-machine interface systems, in which paralysed humans and monkeys learn to control an advanced robotic arm prosthesis directly with their brain activity, via electrodes implanted into the cortex or placed on the scalp [51]–[55]. This work has so far mainly focused on motor control issues and not tackled the problem of how to achieve somatic perception of the artificial limbs. Ehrsson and colleagues have recently demonstrated that upper limb amputees can be induced to experience ownership of a limb prosthesis simply by ‘tricking the mind’ using the multisensory principles from the rubber hand illusion [39], [56]. Thus, body-ownership illusions may contribute to the incorporation of future advanced limb-prostheses into the body-representation. The present results are exiting as they suggest that it could be possible to develop supernumerary limb prostheses. Our findings provide a ‘proof of concept’ that the central nervous system has the capacity to represent more than two upper limbs at the same time. Thus, paralysed patients could experience a supernumerary prosthesis as part of their own bodies while maintaining ownership of their real limbs. Or a person with a functionally impaired arm could perform everyday tasks using a supernumerary arm prosthesis. An obvious important future line of research is to examine how the feeling of ownership of two right hands can be maintained while performing voluntary actions and, even more crucially, whether the motor system can learn to issue independent motor commands to the two owned hands.

In summary, after a period of synchronous visuo-tactile stimulation of a rubber right hand and a person's real right hand both in full view, the rubber hand is experienced as a supernumerary limb. Importantly, this perceptual phenomenon is produced by specific multisensory principles which have been identified in this study in a series of well controlled behavioural and psychophysiological experiments. Thus, under certain circumstances, healthy humans can experience somatic sensations that seem to violate the human body plan.

## Supporting Information

Text S1 The statistical analysis of the illusion-related statements and the control statements in experiment 1.
(DOC)

Text S2 The statistical analysis of the illusion-related statements and the control statements in experiment 3.
(DOC)
