# Supplementary material for: The Illusion of Owning a Third Arm
Source: PLoS One. 2011 Feb 23;6(2):e17208. doi: 10.1371/journal.pone.0017208 (PMC3044173; doi:10.1371/journal.pone.0017208)
Supplement: Text S1 — The statistical analysis of the illusion-related statements and the control statements in experiment 1. (DOC) [file pone.0017208.s001.doc]

**Experiment 1 - Control vs. Illusion statements**

We compared the illusion-related statements (S1-S2 and S5-S6) and the control statements (S7-S10) to see if the difference in ratings was greater in the synchronous (illusion) condition than in the control ones, by using a 2x3 repeated measures ANOVA with the main factors being Statement type (Illusion, Control) and Condition (Synchronous, Asynchronous, Rotated). We observed significant effects of the main factors Statement type (F(1, 119) = 66.558, p < 0.001) and Condition (F(2, 238) = 44.766, p < 0.001). Crucially, a significant interaction Statement type x Condition (F(2, 238) = 26.857, p < 0.001) was found, meaning that the difference between the illusion statements and control statements was greatest in the synchronous conditon. We also compared the synchronous vs. asynchronous and synchronous vs. rotated rubber hand conditions individually, by performing two 2x2 repeated measures ANOVAs using the main factors Statement type (Illusion, Control) and Condition (Synchronous, Asynchronous and Synchronous, Rotated, respectively). All main effects were significant (p < 0.001) and, importantly, both interactions Statement type x Condition (Synchronous, Asynchronous) (F(1, 119) = 52.810, p < 0.001) and (Synchronous, Rotated) (F(1, 119) = 18.842, p < 0.001) were significant. This means that higher scores were obtained for the illusion statements in relation to the control ones during the synchronous condition than for the asynchronous and rotated rubber hand control conditions, respectively.
