# Supplementary material for: The Illusion of Owning a Third Arm
Source: PLoS One. 2011 Feb 23;6(2):e17208. doi: 10.1371/journal.pone.0017208 (PMC3044173; doi:10.1371/journal.pone.0017208)
Supplement: Text S2 — The statistical analysis of the illusion-related statements and the control statements in experiment 3. (DOC) [file pone.0017208.s002.doc]

**Experiment 3 - Control vs. Illusion statements**

We compared the illusion statements to the control ones to see if the difference in ratings was greater in the rubber right hand (illusion) condition than in the control ones. For this, we used a 2x3 repeated measures ANOVA with the main factors Statement type (Illusion, Control) and Condition (Rubber right hand, Rubber left hand, Rubber right foot). Both main factors attained significance (p < 0.001), and, crucially, there was a significant interaction between Statement type x Condition (F(2, 198) = 30.500, p < 0.001), meaning that the difference in the rating scores between the illusion and control statements was greatest for the rubber right hand conditon. To address the difference between the rubber right hand condition and each control condition separately, we performed two 2x2 repeated measures ANOVAs using the main factors Statement type (Illusion, Control) and Condition (Rubber right hand, Rubber left hand and Rubber right hand, Rubber right foot, respectively). All of the main effects in both ANOVAs were significant (p < 0.001) and, importantly, both interactions Statement type x Condition (Rubber right hand, Rubber left hand) (F(1, 99) = 6.110, p = 0.015) and (Rubber right hand, Rubber right foot) (F(1, 99) = 61.357, p < 0.001) were significant. In plain English, this means that higher scores were obtained for the illusion statements in relation to the control ones during the rubber right hand condition (illusion) than for the rubber left hand and the rubber right foot control conditions, respectively.

A complementary analysis was conducted to compare the rating scores for the two control conditions. We decided to do this because the rubber left hand control condition yielded unexpectedly high scores for the “duplication of touch" statement (see above). Thus, in a post-hoc approach, we compared the rubber left hand and rubber right foot conditions using three 2x2 repeated measures ANOVAs with the main factors Limb (Rubber left hand, Rubber right foot) and Statement (Levels being the two statements of the pair in question, as in previous analyses). Analysing the main effect of the factor Limb revealed significantly stronger rubber limb ownership in the rubber left hand condition (S1-S2) (F(1, 24) = 30.147, p < 0.001), no significant difference in disownership (S3-S4) (F(1, 24) = 0.723, p = 0.403), and a significantly stronger feeling of owning two right hands (S5-S6) (F(1, 24) = 18.832, p < 0.001) in the rubber left hand than in the rubber right foot condition. Thus, it seems as if the foot condition is the most powerful at abolishing the illusion.
